# Exit from Pluripotency Is Gated by Intracellular Redistribution of the bHLH Transcription Factor Tfe3

**DOI:** 10.1016/j.cell.2013.03.012

**Published:** 2013-04-11

**Authors:** Joerg Betschinger, Jennifer Nichols, Sabine Dietmann, Philip D. Corrin, Patrick J. Paddison, Austin Smith

**Affiliations:** 1 Wellcome Trust—Medical Research Council Stem Cell Institute, University of Cambridge, Cambridge CB2 1QR, UK; 2 Department of Biochemistry, University of Cambridge, Cambridge CB2 1QR, UK; 3 Department of Physiology, Development and Neuroscience, University of Cambridge, Cambridge CB2 1QR, UK; 4 Human Biology Division, Fred Hutchinson Cancer Research Centre, Seattle, WA 98109, USA

## Abstract

Factors that sustain self-renewal of mouse embryonic stem cells (ESCs) are well described. In contrast, the machinery regulating exit from pluripotency is ill defined. In a large-scale small interfering RNA (siRNA) screen, we found that knockdown of the tumor suppressors *Folliculin* (*Flcn*) and *Tsc2* prevent ESC commitment. Tsc2 lies upstream of mammalian target of rapamycin (mTOR), whereas Flcn acts downstream and in parallel. Flcn with its interaction partners Fnip1 and Fnip2 drives differentiation by restricting nuclear localization and activity of the bHLH transcription factor Tfe3. Conversely, enforced nuclear Tfe3 enables ESCs to withstand differentiation conditions. Genome-wide location and functional analyses showed that Tfe3 directly integrates into the pluripotency circuitry through transcriptional regulation of *Esrrb*. These findings identify a cell-intrinsic rheostat for destabilizing ground-state pluripotency to allow lineage commitment. Congruently, stage-specific subcellular relocalization of Tfe3 suggests that Flcn-Fnip1/2 contributes to developmental progression of the pluripotent epiblast in vivo.

## Graphical abstract

**Graphical Abstract:**

**Figure:**
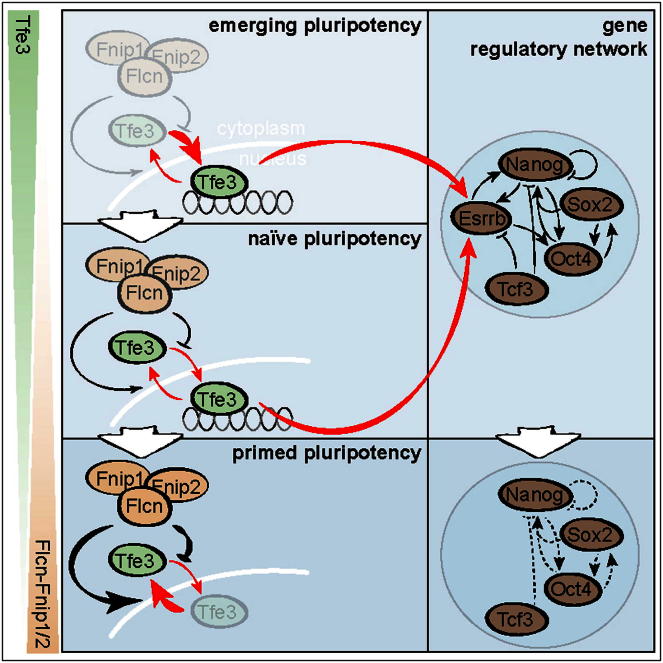

**Highlights:**

► A large-scale siRNA screen identifies drivers of exit from ground-state pluripotency ► Tsc1/2 and Flcn-Fnip1/2 promote ESC commitment via nuclear exclusion of Tfe3 ► Nuclear Tfe3 confers autonomous ESC self-renewal ► Tfe3 directly regulates transcription of the core pluripotency factor Esrrb

## Teaser

An siRNA screen in mouse embryonic stem cells uncovers a signaling axis at the onset of differentiation that stimulates nuclear export of Tfe3, a transcription factor that regulates the pluripotent transcription factor network. This study highlights subcellular compartmentalization of cell-state regulators as a means of enabling cell-fate transition.

## Introduction

Stem cell fate is determined by a balance between pro-self-renewal and pro-differentiation signals. To initiate differentiation, stem cells have to be forced out of self-renewal, the transcriptional networks conferring stem cell identity need to be dissolved, and lineages have to be chosen. How these interdependent tasks are coordinated is poorly understood. Mouse embryonic stem cells (ESCs) provide a tractable system for dissecting this process because intrinsic and extrinsic signals regulating self-renewal are reasonably well defined.

Genetic and biochemical studies have shown that activity of mitogen-activated protein kinase (MAPK) (Kunath et al., 2007) and glycogen synthetase kinase-3 (GSK3) (Doble et al., 2007) prime ESCs for differentiation. Chemical inhibition of both (2i) promotes robust self-renewal (Ying et al., 2008). It is therefore hypothesized that shielding ESCs from differentiation cues is sufficient for stabilization and propagation of a naive pluripotent ground state (Nichols and Smith, 2009).

Pluripotency is governed by a transcription factor network that contains numerous autoregulatory loops, suggestive of self-propagation (Jaenisch and Young, 2008). How this recursive circuitry is extinguished in an orderly manner to enable developmental progression and eventual lineage commitment is currently unclear. Endogenous repressors Tcf3 (encoded by *Tcf7l1*) and the NuRD complex (Kaji et al., 2006; Nichols and Smith, 2012; Pereira et al., 2006) play a role, but other key drivers of exit from naive pluripotency have yet to be identified.

Delineation of mechanisms that steer ESCs into differentiation may reveal predetermined breaking points in the pluripotent transcription factor network. Furthermore, factors regulating ESC fate transition are candidates to drive progression of the naive preimplantation into the lineage-poised postimplantation epiblast in vivo (Nichols and Smith, 2009).

Here, we exploited the ground-state culture system to screen functionally for genes promoting exit from naive pluripotency.

## Results

### A Large-Scale siRNA Screen Identifies Genes Required for Exit from Ground-State Pluripotency

**Figure 1:**
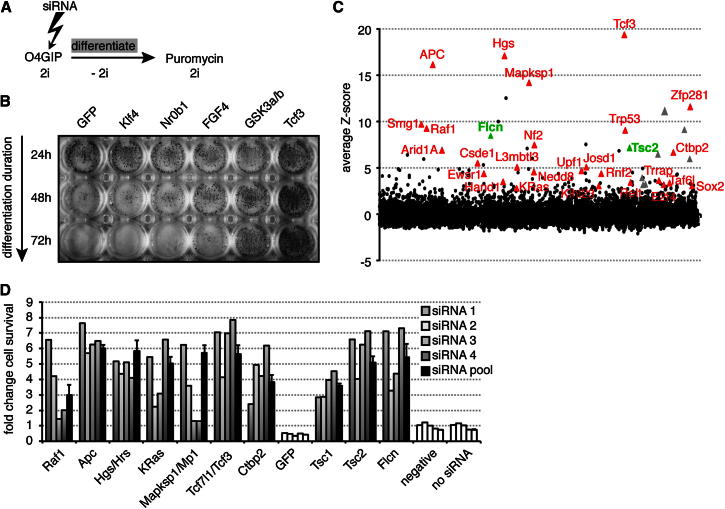
A Large-Scale siRNA Screen for Genes Regulating ESC Commitment (A) Outline of the screening procedure; O4GIP ESCs were transfected with siRNAs in 2i, differentiation enabled by inhibitor removal, and resistance to commitment assayed by restoring 2i with puromycin selection for *Oct4* expression. (B) Exit from pluripotency in differentiating O4GIP ESCs transfected with indicated siRNAs assayed after 24 hr, 48 hr, and 72 hr and stained for AP. (C) Average screen Z scores. Red and green triangles show validated hits (see Figure S1B); gray triangles show duplicates within the transcription factor subset. (D) O4GIP ESC resistance to commitment after transfection with siRNAs was quantified with a cell viability assay and normalized to no siRNA transfection controls. Pools and individual siRNAs are shown. Note that *Tsc1* was not recovered in the primary screen. For siRNA pools, the average and standard deviation (SD) of two technical replicates is shown. See also Figure S1.

To uncover genes that are required for progression from naive pluripotency into a state primed for differentiation, we used a simple monolayer differentiation protocol (Ying et al., 2003). Efficient loss of ESC identity over 3 days (Figures 1A and 1B) is a time window compatible with perdurance of transient knockdown by siRNA. We assayed for resistance to exit the ESC state by ability to proliferate in selective 2i culture conditions plus retained expression of the *Oct4* locus. After transfecting siRNAs in 2i, differentiation was enabled by withdrawal of the inhibitors. Resistance to commitment was then assayed by reapplying inhibitors and selecting for *Oct4* expression (Figure 1A). After 72 hr, cells transfected with *GFP*, *Klf4*, or *Nr0b1* siRNAs lost ESC properties, but upon knockdown of *FGF4*, *GSK3a/b*, or *Tcf3*, significant numbers of alkaline phosphatase (AP) expressing ESCs were recovered (Figure 1B).

**Figure S1:**
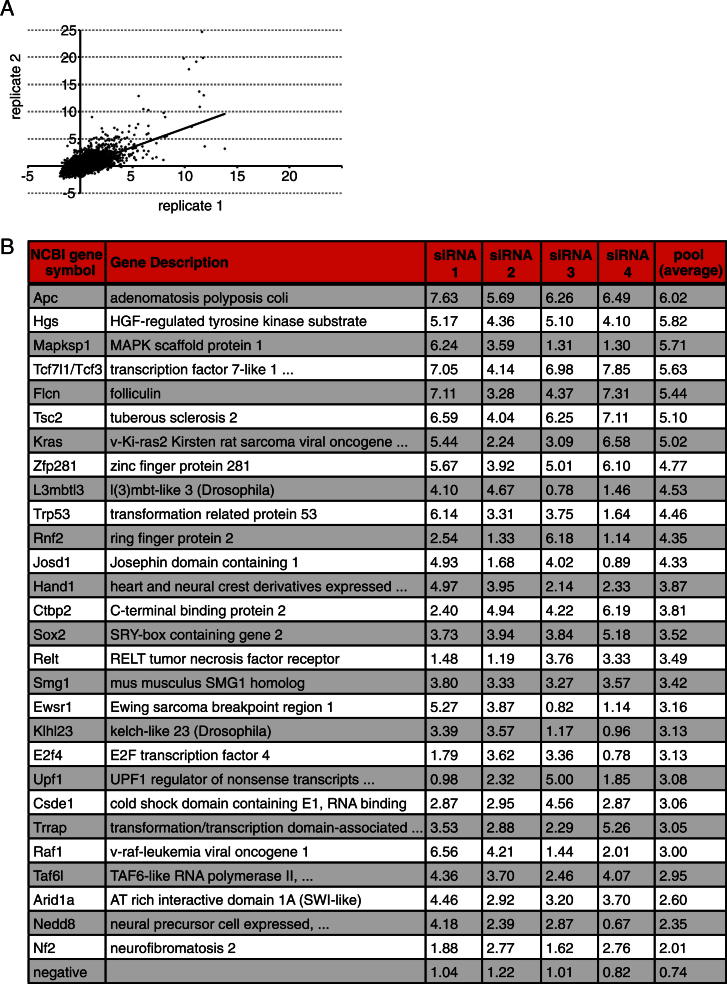
Related to Figure 1 (A) Scatter plot of Z scores for the two replicates. Coefficient of determination R^2^ = 0.483. (B) Validated screen hits. Relative fold increase in cell viability normalized to no siRNA after 72 hr differentiation of O4GIP ESCs transfected with indicated siRNAs. Genes are ranked according to the average of two technical replicates using siRNA pools. An increase greater than 2 is considered to be significant.

To allow quantitation at high throughput, commitment resistance was measured by cell survival. We screened roughly 9,900 genes in duplicate experiments with pools of four independent siRNAs. Z scores were determined for each run (Figure 1C and Figure S1A available online). Seventy genes that stringently scored positive (Z > 3 and Z > 2.5 in the two trials) were intersected with RNA-sequencing data from ESC differentiations (T. Kalkan and A.S., unpublished data), and 17 genes were excluded due to lack of expression. Another 7 genes were manually eliminated as likely false-positives (see Extended Experimental Procedures). The remaining 46 primary hits were retested, and 28 validated with at least two different siRNAs (Figure S1B). These include *Tcf3* and other members of the pathways inhibited in 2i culture conditions (Figure 1D), indicating that the screen successfully identified genes regulating exit from ESC pluripotency.

### *Folliculin* Regulates Exit from Pluripotency

**Figure 2:**
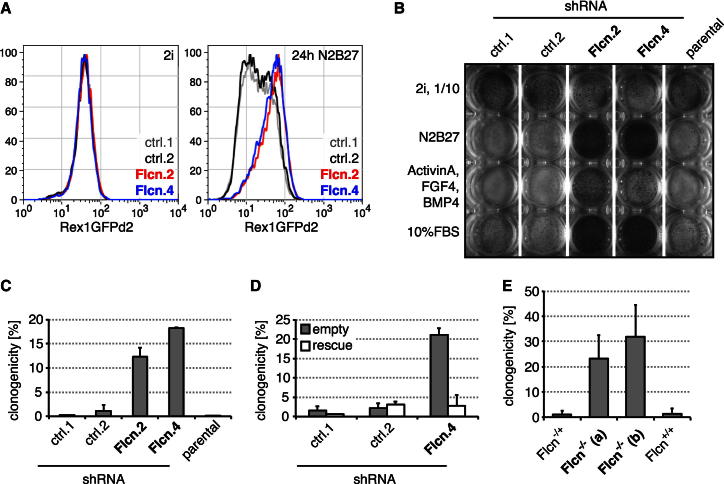
*Flcn* Regulates ESC Commitment (A) Flow cytometry profile of Rex1GFPd2 expression in *Flcn* shRNA knockdown clones (Flcn.2,4) and controls (ctrl.1,2) in 2i conditions (left panel) and after 24 hr of 2i withdrawal (right panel). (B) *Flcn* shRNA cell lines and controls kept in 2i (10-fold fewer cells) or differentiated for 72 hr in N2B27, or N2B27 supplemented with 10% FBS or 25 ng/ml FGF4, 10 ng/ml BMP4, and 20 ng/ml Activin A, were replated in 2i and selected in blasticidin for Rex1 expression. Resulting ESC colonies were visualized by AP staining. (C and D) Differentiating *Flcn* shRNA cell lines (C) or *Flcn* shRNA cell lines expressing an empty vector or shRNA-resistant *Flcn* transgene (rescue) (D) and respective controls were replated at clonal density, and colonies arising from uncommitted cells stained for AP. Average clonogenicity and SD are relative to number of plated cells of two independent experiments. (E) CreERT2-expressing clones of indicated genotypes were treated with Tam, differentiated for 3 days, and uncommitted cells quantified in 2i/LIF. Average and SD are of at least three independent biological replicates. See also Figure S2.

**Figure S2:**
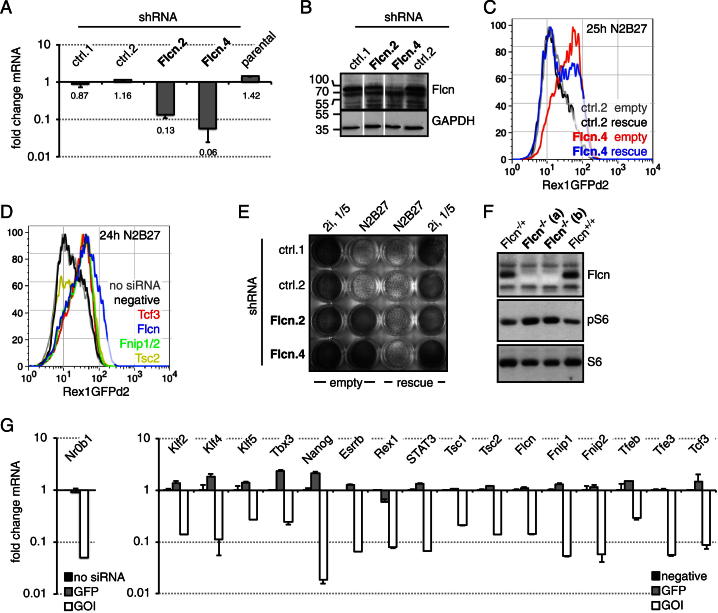
Related to Figure 2 (A) Quantification of *Flcn* mRNA in *Flcn* shRNA cell lines (Flcn.2,4) relative to control (ctrl.1,2) and parental cells. Average and SD are from two technical replicates. (B) Western blot of *Flcn* shRNA knockdown clones and corresponding controls probed for Flcn and GAPDH as a loading control. (C) Maintenance of Rex1GFPd2 expression in *Flcn* shRNA knockdown clones transfected with an empty vector or a plasmid expressing an shRNA-resistant *Flcn* transgene (rescue) after 25 hr of 2i withdrawal. (D) Maintenance of Rex1GFPd2 expression 24 hr after inhibitor withdrawal upon transfection of indicated siRNAs. (E) *Flcn* shRNA knockdown clones and controls expressing an empty vector or the rescue transgene were differentiated for 80 hr or kept in 2i, replated in 2i with Rex1-expression selection, and stained for AP. Note: five times fewer cells were plated for the 2i condition. (F) ESCs wild-type, heterozygous, or homozygous for a floxed *Flcn* allele were stably transfected with CreERT2, and single clones expanded. Cell lines were exposed to 0.1 μM Tam for 48 hr, and cell lysates probed with indicated antibodies. (G) Knockdown efficiencies of utilized siRNAs. ESCs were transfected with indicated siRNAs (GOI: gene of interest), and corresponding mRNA changes calculated relative to negative (or no siRNA) controls. Average and SD are of two technical replicates.

We focused on the tumor suppressor *Folliculin* (*Flcn*) (Nickerson et al., 2002) because a role in pluripotency has not been described, but homozygous knockout alleles show early embryonic lethality (Chen et al., 2008a; Hasumi et al., 2009). Rex1GFPd2 ESCs that quantitatively report differentiation (Marks et al., 2012; Wray et al., 2011; Yang et al., 2012) were engineered to express a small hairpin RNA (shRNA) targeting *Flcn*, resulting in reduced transcript and protein (Figures S2A and S2B). Twenty-four hours after 2i withdrawal, control cells downregulated GFP, whereas *Flcn* shRNA knockdown lines maintained expression (Figure 2A). This phenotype could be partially reverted by a *Flcn* transgene designed to be shRNA resistant (Figure S2C). Maintenance of GFP expression at 24 hr without 2i was also observed after transient knockdown with siRNA transfection (Figures S2D and S2G). Replating differentiating control cells after 3 days into 2i conditions with Rex1-expression selection gave very few AP-positive colonies, whereas *Flcn* knockdown cells yielded many (Figure 2B). We used colony-forming assays to quantify 10%–15% of cells in *Flcn* shRNA knockdown cell lines as uncommitted 3 days after inhibitor withdrawal (Figure 2C). This is a conservative estimate because cloning efficiency of ESCs is typically only 50% (Wray et al., 2010). The phenotype could be rescued with the transgene (Figures 2D and S2E). Resistance to differentiation was also observed in the presence of Activin A, fibroblast growth factor 4 (FGF4), and bone morphogenetic protein 4 (BMP4) or 10% fetal bovine serum (Figure 2B), suggesting that *Flcn* is important for exit from pluripotency under different inductive regimes.

To confirm these findings in a genetic null background, we derived ESCs from *Flcn*^flox/+^ intercrosses (Hasumi et al., 2009). Cells were stably transfected with a plasmid expressing Cre fused to the ligand-binding domain of the estrogen receptor (ERT2), and single-cell clones expanded. After treatment with 4-Hydroxytamoxifen (Tam) to activate Cre, Flcn protein was undetectable in two independently derived knockout ESC lines (Figure S2F). After 3 days of differentiation, wild-type and heterozygous cells lost colony-forming ability, whereas 20%–30% of the knockout cells generated undifferentiated colonies (Figure 2E). These observations confirm that Flcn expedites exit from the ESC state.

### Folliculin Acts downstream of, or in Parallel to, mTOR and Together with Fnip1/2

**Figure 3:**
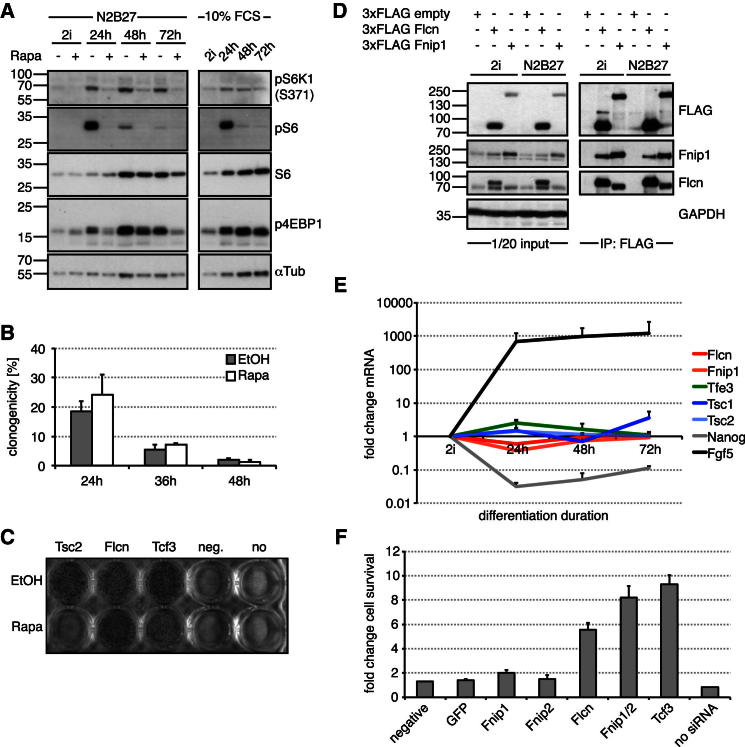
*Flcn* Acts downstream of or in Parallel to mTOR and Interacts with Fnip1/2 (A) Rex1GFPd2 cells were differentiated in N2B27 with and without 20 nM Rapa (left panel) or in N2B27/10%FBS (right panel), and cell lysates probed with indicated antibodies. (B) Rex1GFPd2 cells differentiated in N2B27 with and without 20 nM Rapa were replated at single-cell density in 2i including Rex1-expression selection at the indicated time points. The average percentage of uncommitted cells forming AP-positive colonies relative to the number of cells plated and SD are of two technical replicates. (C) Rex1GFPd2 cells transfected with indicated siRNAs were differentiated for 72 hr in N2B27 with and without 20 nM Rapa and replated in 2i with Rex1-expression selection, and resulting colonies were stained for AP. (D) Proteins were immunoprecipitated with FLAG antibodies from stably transfected Rex1GFPd2 cells cultured in 2i or differentiated for 40 hr and probed with indicated antibodies. (E) mRNA levels were quantified during differentiation and normalized to 2i-cultured cells. Average and SD are of two cell lines. (F) O4GIP ESCs were transfected with indicated siRNAs, and after differentiation for 3 days, exit from pluripotency quantified with a cell-viability assay and normalized to no siRNA transfection controls. Average and SD are of two technical replicates. See also Figure S3.

**Figure S3:**
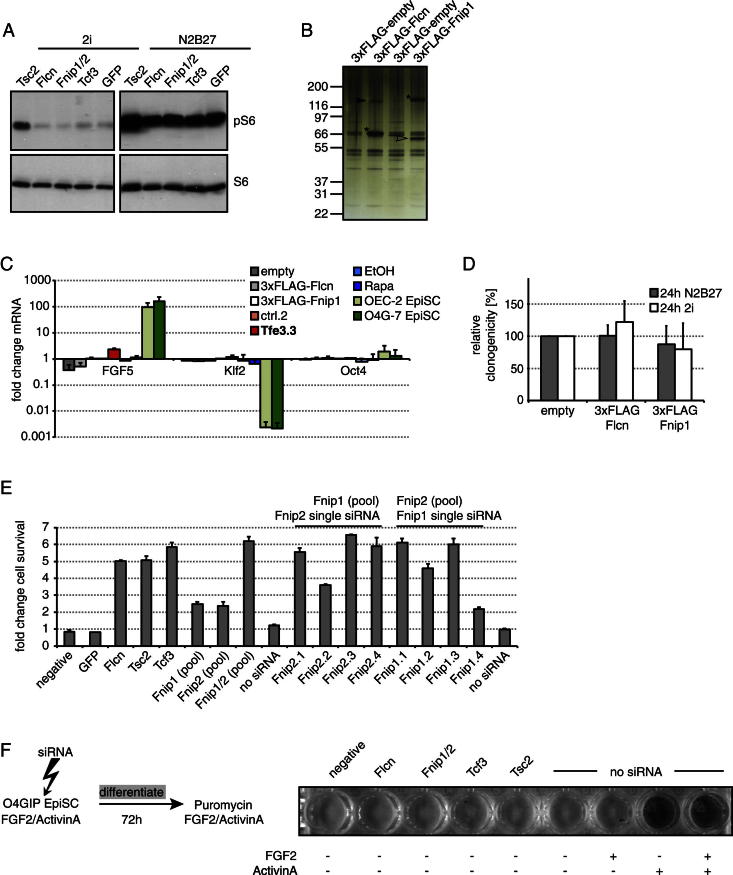
Related to Figure 3 (A) ESCs were transfected with siRNAs overnight, and after 36 hr in indicated culture conditions, cell lysates were probed with specified antibodies. Equal exposures indicate that the increase in S6 phosphorylation during differentiation exceeds the effect of *Tsc2* depletion in undifferentiated ESCs. (B) Silver-stained gel of FLAG-IP eluates from ESCs expressing indicated constructs. Bands corresponding to the IP’ed protein are marked with an asterisk. FLAG-Flcn co-IPs Fnip1 (arrowhead) and FLAG-Fnip1 co-IPs a band at the size of Flcn (open arrowhead). No other specific bands are visible compared to empty vector controls. (C) Fold changes of indicated transcript levels in ESCs overexpressing the Flcn-Fnip1 complex (empty, 3xFLAG-Flcn, 3xFLAG-Fnip1), stably knocked down for Tfe3 (empty shRNA [ctrl.2], Tfe3 shRNA [Tfe3.3]) or depleted of mTOR activity by rapamycin (EtOH, Rapa) relative to empty shRNA (ctrl.2). For comparison, two O4GIP EpiSC lines are included. Average and SD are of two independent experiments. (D) ESCs overexpressing the Flcn-Fnip1 complex (empty, 3×FLAG-Flcn, 3×FLAG-Fnip1) were exposed for 24 hr to the indicated culture conditions, and ESCs quantified by replating single cells in 2i with Rex1-expression selection. Average clonogenicity relative to empty vector control and SD are of four independent experiments. (E) Deconvolution of Fnip1/2 siRNA pools. O4GIP ESCs were transfected with indicated siRNAs, and after 3 days of differentiation, exit from pluripotency quantified with a cell-viability assay and normalized to no siRNA transfection controls. Average and SD are of two technical replicates. (F) O4GIP-7 EpiSCs were transfected with indicated siRNAs and differentiated by removal of FGF2 and Activin A for 3 days. After reapplication of EpiSC culture conditions and Oct4-expression selection using puromycin, EpiSCs were visualized by AP activity.

Flcn has been linked to mammalian target of rapamycin (mTOR) kinase signaling (Baba et al., 2006; Hartman et al., 2009; Piao et al., 2009). The mTOR inhibitor *Tsc2* scored as a commitment regulator in our screen (Figure 1D). Tsc2 functions as a heterodimer with Tsc1 (Laplante and Sabatini, 2012), and knockdown of *Tsc1* also inhibited exit from pluripotency (Figure 1D). However, we found that mTOR was strongly activated after inhibitor withdrawal, leading to increased phosphorylation of ribosomal protein S6 (S6), S6kinase, and the translational regulator 4EBP1 (Figure 3A). The phosphorylation kinetics differed; in particular, S6 and 4EBP1 phosphorylation peaked at 24 hr and 48 hr, respectively. These effects are not due to starvation in N2B27 as they were also observed in serum. To test whether activation of mTOR is important for ground-state exit, we quantified undifferentiated ESCs at 24 hr, 36 hr, and 48 hr of 2i withdrawal in the presence or absence of the mTOR inhibitor rapamycin (Rapa) (Figure 3B). No difference was detected. As expected, knockdown of *Tsc2* increased S6 phosphorylation (Figure S3A) and impaired Rex1GFPd2 downregulation (Figure S2D). Blocking mTOR with Rapa in this context abrogated the differentiation defects (Figure 3C). These data indicate that elevated activity of mTOR in ESCs impedes exit from self-renewal, but that activation during differentiation is not required for cell-fate transition. Rapa did not affect *Tcf3* or *Flcn* knockdown phenotypes (Figure 3C), suggesting that Flcn acts downstream or independent of mTOR.

Flcn has been reported to bind to two Flcn-interacting proteins, Fnip1 and Fnip2 (Baba et al., 2006; Hasumi et al., 2008; Takagi et al., 2008). To identify binding partners in ESCs, we immunopurified (IP) Flcn from cells expressing functional 3×FLAG-Flcn (data not shown). Silver staining revealed the presence of one specific copurifying protein at approximately 130 kDa that was identified as Fnip1 by mass-spectrometry (Figure S3B, arrowhead). Vice versa, 3×FLAG-Fnip1 copurified one specific protein with a molecular weight similar to that of Flcn (Figure S3B, open arrowhead). Endogenous Fnip1 and Flcn were detected in 3×FLAG-Flcn and 3×FLAG-Fnip1 IPs, respectively (Figure 3D), confirming the presence of a Flcn-Fnip1 protein complex in ESCs.

*Flcn*, *Tsc1*, *Tsc2*, and *Fnip1* messenger RNA (mRNA) levels did not change markedly during early differentiation as compared to downregulation of *Nanog* and upregulation of *FGF5* (Figure 3E). Furthermore, Flcn or Fnip1 overexpression did not induce transcriptional changes indicative of differentiation (Figure S3C) or accelerate ESC commitment (Figure S3D). This suggests that Flcn and Fnip1 levels are not limiting for exit from pluripotency.

To test the functional significance of the Flcn-Fnip1 interaction, we knocked down *Fnip1* and its homolog *Fnip2* and assayed loss of ESC identity after 2i withdrawal. Although neither *Fnip1* nor *Fnip2* single knockdown had a strong impact, knockdown of both had a comparable effect to loss of *Flcn* (Figures 3F and S3E). Consistent with this, Rex1GFPd2 cells transfected with *Fnip1/2* siRNAs retained GFP expression after 24 hr of withdrawal from 2i (Figure S2D).

To investigate whether the functions of mTOR and Flcn in regulation of pluripotency are ESC specific, we turned to postimplantation epiblast stem cells (EpiSCs) (Brons et al., 2007; Tesar et al., 2007). EpiSCs express *Oct4*, *Flcn*, *Fnip1/2*, *Tcf3*, and *Tsc1/2* (Hayashi et al., 2011). O4GIP EpiSCs were induced to differentiate for 3 days through withdrawal of growth factors and assayed for commitment by reapplying EpiSC culture conditions and selecting for *Oct4* expression. Neither *Tcf3*, *Tsc2*, *Flcn*, nor *Fnip1/2* knockdown impaired EpiSC differentiation (Figure S3F), suggesting that the role of these factors is specific to the cell context.

### Flcn Regulates *Tfe3* Localization and Activity

**Figure 4:**
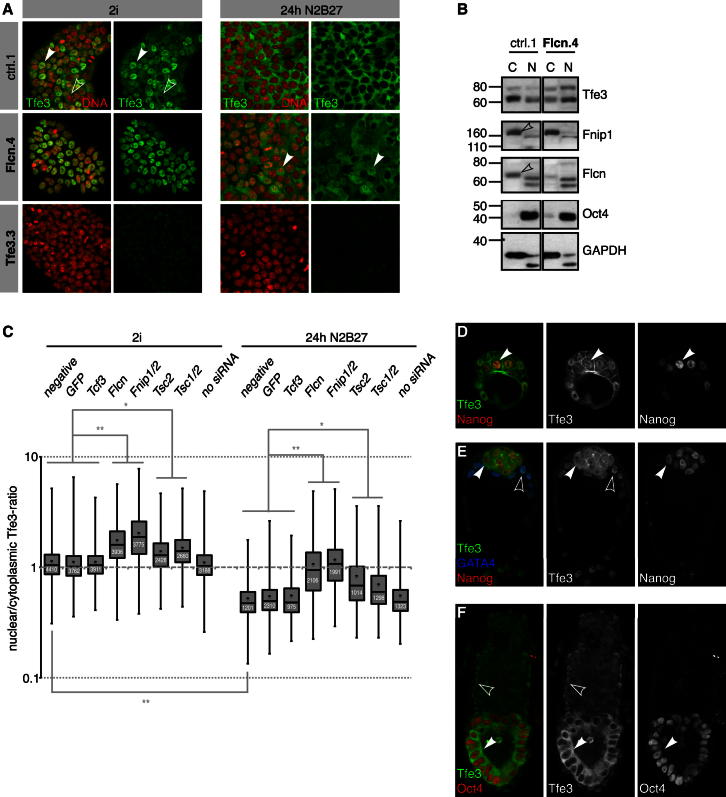
*Flcn* Regulates Subcellular Localization of Tfe3 (A) Control (ctrl.1), *Flcn* shRNA (Flcn.4), and *Tfe3* shRNA (Tfe3.3) cell lines were stained for Tfe3 and DNA in 2i conditions (left panel) and 24 hr after inhibitor withdrawal (right panel). Tfe3 was detected in the nucleus (arrowhead) and cytoplasm (open arrowhead). (B) Cytoplasmic (C) and nuclear (N) fractions of control and *Flcn* shRNA cells probed with indicated antibodies (open arrowheads indicate Fnip1 and Flcn bands; both bands recognized by Tfe3 antibodies are specific and likely represent phosphorylation variants; Hong et al., 2010). (C) Box and whisker plots of nuclear/cytoplasmic Tfe3 ratios in ESCs transfected with indicated siRNAs in 2i conditions and 24 hr after inhibitor withdrawal. Indicated cell numbers (white) from three experiments with two ESC lines were quantified. (^∗∗^) and (^∗^) indicate Student’s t test values < 1 × 10^−100^ and 1 × 10^−50^, respectively. (D–F) Tfe3 is localized to the nucleus (arrowhead) at E3.5 (D). At E4.5, Tfe3 is found in the nucleus and cytoplasm of Nanog-positive epiblast cells (arrowhead) but stays nuclear in GATA4-positive (open arrowhead) primitive endodermal cells (E). At E5.5, Tfe3 is enriched in the cytoplasm (arrowhead) of Oct4-positive epiblast cells and remains nuclear (open arrowhead) in extraembryonic endoderm cells (F). See also Figure S4.

**Figure S4:**
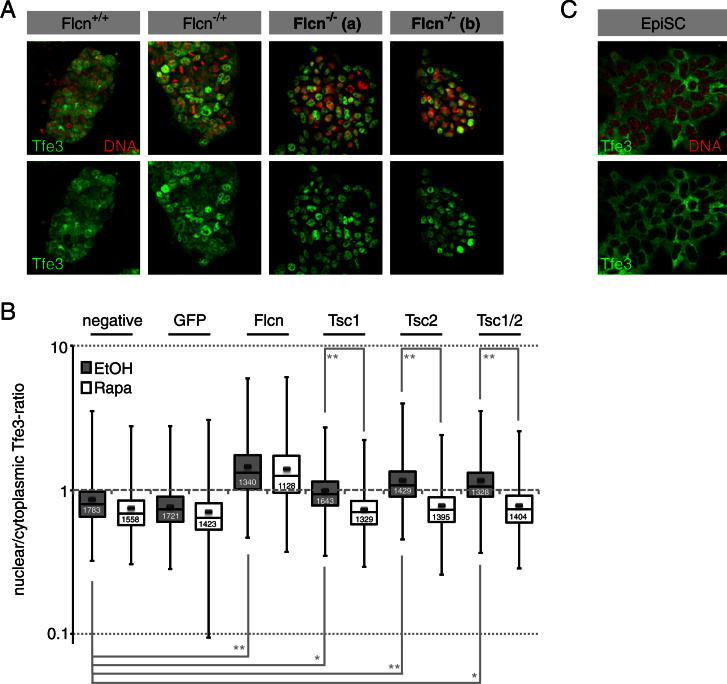
Related to Figure 4 (A) Subcellular Tfe3 localization in wild-type, heterozygous, and homozygous *Flcn* knockout clones. (B) Box and whisker plots of nuclear/cytoplasmic ratios of ESCs transfected with indicated siRNAs in 2i in the presence and absence of 0.1 μM Rapa. Indicated cell numbers (white) were quantified. (^∗∗^) and (^∗^) indicate Student’s t test values < 1 × 10^−100^ and 1 × 10^−25^, respectively. Note that absolute values of nuclear/cytoplasmic ratios are different to Figure 4C due to independent experiments and quantitation. (C) Tfe3 localization in O4GIP EpiSCs.

Flcn regulates nuclear translocation of the bHLH transcription factor Tfe3 in human kidney cells and mouse embryonic fibroblasts (Hong et al., 2010). Tfe3 showed heterogeneous subcellular localization in ESCs in 2i and could be detected in the nucleus (Figure 4A, left panel, arrowhead), cytoplasm (Figure 4A, left panel, open arrowhead), or both compartments. Staining was specific as it was reduced in *Tfe3* shRNA knockdown cells (Figure 4A, lower panel). *Tfe3* mRNA levels did not change distinctly during ESC differentiation (Figure 3E), but we observed a marked cytoplasmic relocalization (Figure 4A, right panel). In contrast, most *Flcn* shRNA cells featured exclusively nuclear Tfe3 in 2i (Figure 4A, left panel), and many retained nuclear protein after inhibitor withdrawal (Figure 4A, right panel, arrowhead). Consistently, biochemical nucleocytoplasmic fractionations showed more Tfe3 in the nuclear fraction of *Flcn* shRNA knockdown cells compared to control ESCs (Figure 4B). Tfe3 was also enriched in the nuclei of *Flcn* null ESCs (Figure S4A). We used automated image quantification to determine nuclear-cytoplasmic ratios of *Tfe3* in siRNA-treated cells (see Extended Experimental Procedures). Compared to controls, knockdown of *Flcn* and *Fnip1/2* lead to a significant increase in nuclear Tfe3 both in 2i conditions and 24 hr after inhibitor withdrawal (Figure 4C). Importantly, this was not an indirect consequence of maintained ESC identity, as *Tcf3*-depleted cells showed cytoplasmic Tfe3. Knockdown of *Tsc2* increased nuclear Tfe3 concentration in an mTOR-dependent manner (Figures 4C and S4B), although less pronounced than after knockdown of *Flcn* or *Fnip1/2*. These observations suggest that Flcn-Fnip1/2 and Tsc2 activities converge on subcellular localization of Tfe3 and that nuclear exclusion of Tfe3 normally correlates with loss of ground-state pluripotency.

In EpiSCs, Tfe3 is exclusively cytoplasmic (Figure S4C). We therefore examined Tfe3 localization in the embryo. Tfe3 was localized in the nuclei of both inner cell mass (ICM) (Figure 4D, arrowhead) and trophectoderm (TE) cells at embryonic day (E) 3.5. At E4.5, Tfe3 could be detected in nuclei and cytoplasm of Nanog-positive epiblast cells (Figure 4E, arrowhead) but was exclusively nuclear in GATA4-positive hypoblast (Figure 4E, open arrowhead) and TE cells. In postimplantation E5.5 embryos, Tfe3 was strongly expressed but excluded from the nucleus in Oct4-positive epiblast cells (Figure 4F, arrowhead), whereas it remained nuclear in the extraembryonic endoderm (Figure 4F, open arrowhead). Thus, nuclear exclusion of Tfe3 in the epiblast correlates with progression from the naive preimplantation state to the postimplantation egg cylinder.

### Tfe3 Acts in ESC Commitment

**Figure 5:**
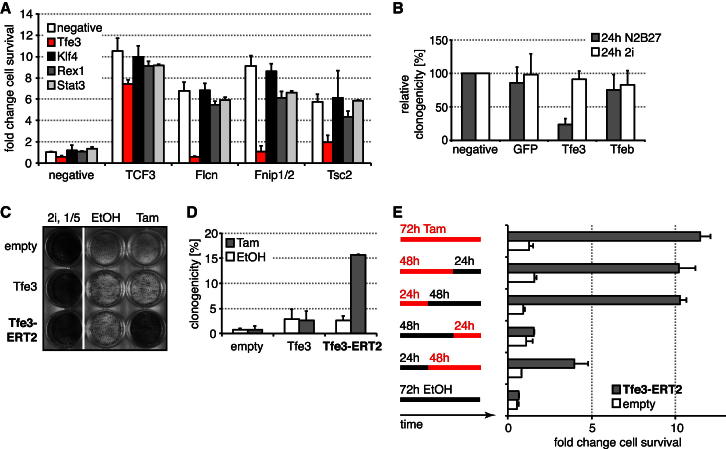
Tfe3 Is Required and Sufficient to Impair ESC Commitment (A) Commitment of O4GIP cells transfected with the indicated siRNA combinations assayed after 72 hr of differentiation by reapplying 2i culture conditions and Oct4-expression selection. Exit from pluripotency was quantified with a cell-viability assay and normalized to negative siRNA treatment. Average and SD are of two technical replicates. (B) Rex1GFPd2 cells were transfected with indicated siRNAs and exposed for 24 hr to the indicated culture conditions, and ESCs were quantified by replating single cells in 2i with Rex1-expression selection. Average clonogenicity relative to negative siRNA and SD are of four independent experiments. (C and D) Rex1GFPd2 cells expressing indicated constructs were differentiated in the absence or presence of 0.1 μM Tam for 100 hr and replated in 2i with Rex1 selection (five times fewer cells were replated for cells kept in 2i), and resulting ESC colonies were visualized with AP (C). Cells differentiated for 72 hr were replated at clonal density, and resulting colonies arising from uncommitted cells stained for AP (D). Average clonogenicity relative to number of plated cells and SD are of two independent experiments. (E) O4GIP ESCs expressing an empty vector or Tfe3-ERT2 were differentiated in the indicated intervals with 0.1 μM Tam and EtOH and switched back to 2i with puromycin selection, and ESCs were quantified with a cell-viability assay. Average fold changes over empty vector control at 72 hr EtOH and SD are from two technical replicates. See also Figure S5.

**Figure S5:**
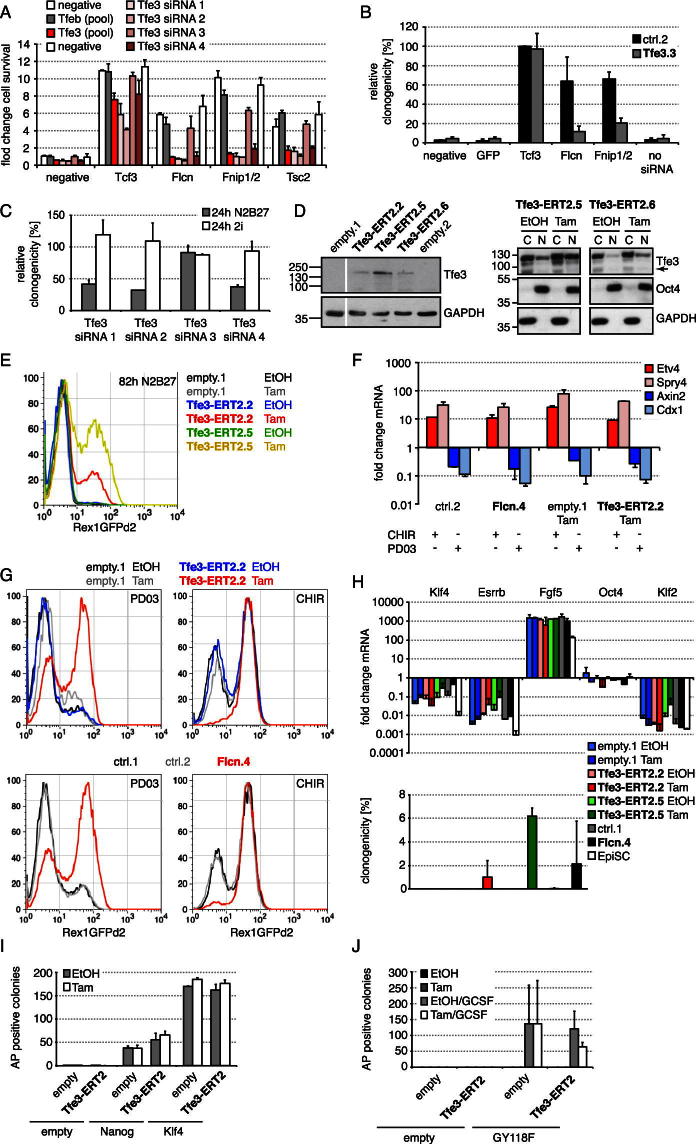
Related to Figure 5 (A) Deconvolution of the *Tfe3* siRNA pool. O4GIP ESCs were transfected with indicated siRNA combinations, and after differentiation for 3 days, exit from pluripotency quantified with a cell-viability assay and normalized to negative siRNA transfection. Average and SD are of two technical replicates. (B) Clonogenicity of differentiated *Tfe3* shRNA knockdown cells (Tfe3.3) and controls (ctrl.2) transfected with indicated siRNAs and replated into 2i with Rex1-expression selection. To account for transfection efficiency variability, resulting AP-positive clone numbers were normalized to *Tcf3* siRNA in control cells. Average and SD are of two independent experiments. (C) Deconvolution of the Tfe3 siRNA pool. Rex1GFPd2 cells were transfected with individual siRNAs and exposed for 24 hr to the indicated culture conditions, and ESCs quantified by replating single cells in 2i with Rex1-expression selection. Average clonogenicity and SD are of two technical replicates relative to negative siRNA-treated controls (Figure 5B). (D) Western blot of Tfe3-ERT2-expressing clones probed with Tfe3 and GAPDH antibodies to control for loading (left panel). Cytoplasmic (C) and nuclear (N) fractions of two Tfe3-ERT2-expressing clones probed with indicated antibodies (right panel). Arrow indicates endogenous Tfe3. (E) Flow cytometry of Rex1GFPd2 expression in Tfe3-ERT2.2 and 5 clones, and empty.1 vector control differentiated in the presence or absence of 0.1 μM Tam for 82 hr. (F) *Flcn* shRNA knockdown (Flcn.4) and Tfe3-ERT2-expressing ESC clones with respective controls were treated for 6 hr in the presence of PD03 or CHIR only and expression of indicated mRNAs determined. Average relative to the respective genotype maintained for 6 hr in 2i and SD are of two independent experiments. (G) Flow cytometry of Rex1GFPd2 expression in Tfe3-ERT2-expressing (upper panels), *Flcn* shRNA knockdown cell clones (lower panels) and controls maintained for four passages in PD03 (left panels) or CHIR (right panels). (H) Tfe3-ERT2-expressing clones (empty.1, Tfe3-ERT2.2 and Tfe3-ERT2.5) were culture for three passages in EpiSC conditions with and without Tam. Similarly, Flcn shRNA knockdown clones (ctrl.1 and Flcn.4) were converted for three passages into EpiSC culture conditions. Fold transcript changes relative to parental cells in 2i and SD are from two technical replicates, and for comparison, expression in O4GIP EpiSCs is shown (upper panel). Exit from the ESC state was quantified by replating single cells in 2i with Rex1-expression selection. Average clonogenicity relative to number of plated cells and SD are of three independent experiments (lower panel). (I and J) OEC-2 EpiSCs coexpressing an empty vector (empty) and Tfe3-ERT2 with reprogramming factors (empty, Nanog or Klf4) (I) or a chimeric LIF-receptor (empty, GY118F) (J) were treated for 4 days with 2i conditions in the presence or absence of Tam, and including GCSF to activate the chimeric receptor (J). Cells were then switched to 2i with selection for Oct4 expression, and emerging ESC colonies quantified by AP. Averages and SD are of two independent experiments.

To test whether Tfe3 is functionally downstream of Flcn-Fnip1/2, we performed simultaneous siRNA transfections. Knockdown of the pluripotency-associated genes *Klf4*, *Rex1*, or *Stat3* did not restore commitment in *Tcf3*-, *Flcn*-, *Fnip1/2*-, or *Tsc2*-depleted cells. However, knockdown of *Tfe3* completely suppressed the *Flcn, Fnip1/2*, and *Tsc2* phenotypes although only mildly affecting *Tcf3*-dependent commitment (Figures 5A and S5A). Conversely, transfection of *Flcn* or *Fnip1/2* siRNAs had little impact in *Tfe3* shRNA knockdown cells (Figure S5B). Thus, *Tfe3* is epistatic to *Flcn*, *Fnip1/2*, and *Tsc2*.

Upon depletion of *Tfe3*, we saw no effect on self-renewal in 2i but a reduction in ESC clonogenicity 24 hr after inhibitor withdrawal (Figures 5B and S5C). Interestingly, knockdown of the Tfe3 family member Tfeb had little effect on its own. We conclude that Tfe3 is not essential for naive pluripotency but constrains exit from ESC self-renewal.

To examine whether nuclear Tfe3 is sufficient to prevent loss of pluripotency, we generated ESCs expressing Tfe3 fused to ERT2 to allow for induction of nuclear translocation by Tam. Tfe3 and Tfe3-ERT2 transfectants were withdrawn from 2i in the absence or presence of Tam and, after 3–4 days, replated in 2i with selection for Rex1 expression. Whereas Tfe3-expressing cells exhibited only mild commitment defects, Tam-induced Tfe3-ERT2-expressing cells showed significantly reduced exit from the ESC state (Figures 5C and 5D). To address the timing of Tfe3 action, we pulsed exposure to Tam. Induction during the first 24 hr after 2i withdrawal was sufficient to impair commitment, whereas later Tam treatment gave weaker phenotypes (Figure 5E). Rex1GFPd2 cells expressing Tfe3-ERT2 maintained GFP after 24 hr in N2B27 in the presence of Tam (data not shown). Even after 76 hr, Rex1GFPd2 expression was maintained in a substantial subset of cells (10%–20%) (data not shown). To test whether this heterogeneous response is due to Tfe3-ERT2 expression variation within the pool of transfectants or due to variable phenotypic penetrance, we picked clones with defined expression levels. Indeed, higher expression and, consequently, more nuclear Tfe3 (Figure S5D) correlated with a higher frequency of GFP maintenance after 3 days of differentiation (Figure S5E). These data are consistent with nuclear Tfe3 inhibiting initial events in the exit from naive pluripotency.

To test for possible crosstalk between the Flcn-Fnip1/2-Tfe3 axis and the differentiation-promoting activities of GSK3 or MAPK signaling, we analyzed downstream targets. FGF4 induces *Etv4* and *Spry4* (Lanner et al., 2010), whereas GSK3 inhibition derepresses *Axin2* and *Cdx1* (Martello et al., 2012; Wray et al., 2011). Upon removal of the respective inhibitor, mRNA levels changed similarly to those in controls in *Flcn* shRNA, *Flcn* knockout, or ectopic nuclear *Tfe3* cell lines (Figure S5F and data not shown). Thus, Tfe3 is unlikely to generally inhibit GSK3 or MAPK signaling. Overlap between pathways regulating ESC self-renewal is well documented (Martello et al., 2012; Nichols and Smith, 2012; Niwa et al., 2009; Wray et al., 2010), and we therefore tested whether *Flcn* and *Tfe3* could compensate for GSK3 or MAPK inhibition. *Flcn* shRNA or ectopic nuclear Tfe3 ESC lines cultured in the presence of either inhibitor alone maintained higher Rex1GFPd2 expression compared to the respective controls even after serial passaging (Figure S5G). This indicates that Flcn-Fnip1/2-Tfe3 operates independently of GSK3 or MAPK signaling as a third module to enable ESC differentiation.

ESC differentiation products can be converted into EpiSCs by continuous exposure to FGF2 and Activin A (Guo et al., 2009). After three passages in these culture conditions, both Tfe3-ERT2-expressing and *Flcn* knockdown cell populations acquired a transcriptional signature similar to that of EpiSCs (Figure S5H, upper panel). However, a small percentage of cells remained clonogenic in 2i (Figure S5H, lower panel), suggesting that nuclear Tfe3 reduces transition into a postimplantation cell fate even in a prolonged and highly inductive context.

Ablation of signals mediating exit from the ESC state during differentiation might also facilitate reprogramming. However, neither depletion of *Flcn*, *Fnip1/2*, or *Tsc2* nor nuclear translocation of Tfe3 enhanced reprogramming of O4GIP EpiSCs into naive pluripotent ESCs, even in sensitized backgrounds overexpressing Klf4 (Guo et al., 2009) and Nanog (Silva et al., 2009), or hyperactive LIF-STAT3 signaling (Yang et al., 2010) (Figures S5I and S5J and data not shown). Thus, Flcn-Fnip1/2-Tfe3 acts specifically in exit from pluripotency during differentiation and not in acquisition of pluripotency during reprogramming.

### Identification of Tfe3 Downstream Targets

**Figure S6:**
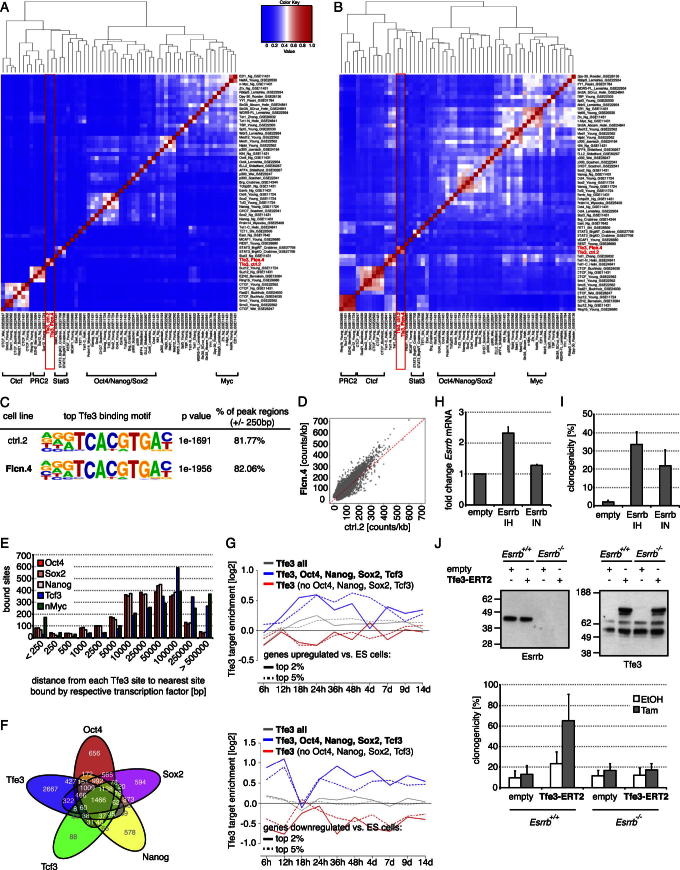
Related to Figure 6 (A and B) Hierarchical clustering of regions (A) and associated genes (B) bound by pluripotency regulators (Martello et al., 2012) and Tfe3 in control (ctrl.2) and *Flcn* shRNA (Flcn.4) ESCs. Colors indicate correlation levels for pairwise comparisons. Factors have been clustered according to correlation level. Cluster groups are indicated in brackets and Tfe3 in a box. (C) Top de novo motif recognition hit for Tfe3 ChIP in control and *Flcn* shRNA cell lines. (D) Read counts in peak regions normalized by the size of the peak region/kB in control and *Flcn* shRNA cells. (E) Distance to the nearest transcription factor for each Tfe3-occupied locus. (F) Venn diagram depicting overlap of genes predicted to be bound by different transcription factors. (G) Enrichment of Tfe3 and Tfe3 together with or exclusive of pluripotency factors at the top 2% or 5% of genes up- (upper panel) or downregulated (lower panel) during ESC differentiation. (H) Overexpression levels of Esrrb mRNA in two ESC lines (Esrrb IH, Esrrb IN) in 2i. Fold change over an empty vector control and SD are of two independent experiments. (I) Exit from the ESC state 80 hr after inhibitor withdrawal was quantified by plating single cells in 2i with Rex1-expression selection. Clonogenicity of indicated genotypes relative to number of cells plated and SD are of three independent experiments. (J) Control and *Esrrb* knockout cells were stably transfected with empty and Tfe3-ERT2-expressing vectors. Expression levels of Esrrb and Tfe3-ERT2 were visualized by western blotting (upper panel). Exit from the ESC state 5 days after differentiation was quantified in single-cell assays (lower panel). Clonogenicity of indicated genotypes relative to number of cells plated and SD are of five independent experiments. Note that cells were propagated in 2i containing LIF to allow self-renewal of *Esrrb* knockout ESCs (Martello et al., 2012), which leads to an increased persistence of ESCs during differentiation as compared to ESCs maintained in 2i alone.

To explore the molecular function of Tfe3 downstream of *Flcn*, we performed chromatin immunoprecipitation coupled to deep sequencing (ChIP-Seq). We identified 6,789 peaks in control and 8,941 peaks in *Flcn* shRNA cell lines. Cluster analysis showed a high overlap between both Tfe3 ChIP data sets (Figures S6A and S6B), and de novo motif recognition identified a modified E box in more than 80% of bound loci in both experiments (Figure S6C). This suggests that Flcn-Fnip1/2 regulates the amount of chromatin-bound Tfe3 rather than binding specificity. Consistent with this, normalized read numbers per peak region were greater in *Flcn* shRNA compared to control cells (Figure S6D).

We compared Tfe3 binding with published ESC ChIP data sets (Martello et al., 2012). Cluster analysis of peak regions (Figure S6A) and associated genes (Figure S6B) showed that the Tfe3-binding profile is distinct and well separated from that of a group containing the bHLH transcription factor n-Myc but closer to the core pluripotency factor family including Oct4, Nanog, and Sox2. The majority of sites occupied by Oct4, Sox2, Nanog, and Tcf3 are distant from sites bound by Tfe3 (Figure S6E). However, a significant fraction of common target genes of Oct4, Sox2, Nanog, and Tcf3 are associated with Tfe3 sites (Figure S6F). This group is more likely transcriptionally up- or downregulated during ESC differentiation compared with genes exclusively bound by Tfe3 (Figure S6G), suggesting a functional relationship between Tfe3 and the pluripotency network.

**Figure 6:**
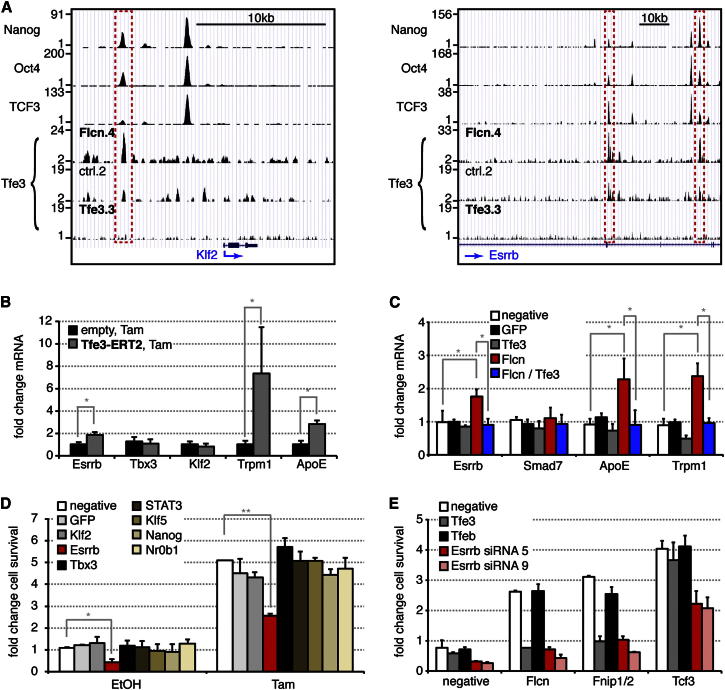
Genome-wide Tfe3-Target Determination Identifies *Esrrb* as a Downstream Effector of Flcn-Fnip1/2-Tfe3 (A) Gene tracks of loci identified by Tfe3 ChIP-Seq with control (ctrl.2), *Flcn* shRNA (Flcn.4), and *Tfe3* shRNA (Tfe3.3) cell lines, overlaid with Nanog-, Oct4- and Tcf3-bound regions. (B) ESCs expressing indicated constructs were treated for 3 hr with 0.1 μM Tam in 2i. Average mRNA fold changes relative to EtOH treatment and SD are from two independent experiments with three different cell lines per genotype. (^∗^) indicates Student’s t test values < 0.005. (C) Average mRNA changes in ESCs transfected with the indicated siRNA combinations. Average relative expression normalized to no and negative siRNA treatments and SD are of three independent experiments. (^∗^) indicates Student’s t test values < 0.05. (D) O4GIP ESCs expressing Tfe3-ERT2 were transfected with indicated siRNAs, differentiated for 3 days in the presence of 0.1 μM Tam or EtOH, and switched back to 2i with puromycin selection, and remaining ESC colonies were quantified with a cell-viability assay. Average fold changes over negative siRNA-transfected, EtOH-treated cells and SD are from two independent experiments. (^∗∗^) and (^∗^) indicate Student’s t test values < 0.001 and 0.03, respectively. (E) Commitment of O4GIP cells transfected with indicated siRNA combinations, including two independent *Esrrb* siRNAs. Exit from pluripotency was quantified with a cell-viability assay and normalized to negative siRNA treatment. Average and SD are of two technical replicates. See also Figure S6.

Because pluripotency transcription factors are strongly relayed onto themselves and exhibit pronounced colocation (Chen et al., 2008b), we examined the overlap of Tfe3, Oct4, Sox2, Nanog, and Tcf3 peaks (Table S1). Co-occupied loci included putative *cis*-regulatory elements in the pivotal pluripotency regulators *Klf2*, *Esrrb* (Figure 6A), and *Tbx3* (data not shown), suggesting that these may be nodes where Tfe3 connects to the pluripotent circuitry.

To assess functionality of Tfe3 binding, we analyzed mRNA levels after 3 hr of Tam addition to Tfe3-ERT2-expressing cells. We included *ApoE* and *Trpm1*, which are bound by Tfe3 (data not shown) and have been described to be regulated by Flcn (Hong et al., 2010) and the Tfe3 homolog MiTF (Miller et al., 2004), respectively. In 2i, we detected induction of *Trpm1* and *ApoE* and a modest but significant upregulation of *Esrrb*, though not *Tbx3* or *Klf2* (Figure 6B). Although only about 2-fold, the upregulation of *Esrrb* is notable on top of the induction by GSK3 inhibition (Martello et al., 2012). In fact, less than 2-fold overexpression of Esrrb was sufficient to impede exit from pluripotency (Figures S6H and S6I). To test whether Flcn regulates this activity of Tfe3, we analyzed target genes after *Flcn* siRNA transfection. Indeed, *Esrrb*, *ApoE*, and *Trpm1* were upregulated after *Flcn* knockdown but not after codepletion of *Tfe3*. In contrast, an unrelated Tfe3-binding target, *Smad7*, was not regulated in ESCs (Figure 6C). These observations suggest that Flcn may drive exit from pluripotency by restricting nuclear access of Tfe3 and consequently reducing transcriptional activation of the core pluripotency factor *Esrrb*.

To test for functional relevance, we performed epistasis experiments and knocked down *Esrrb*, *Klf2*, *Tbx3*, and other pluripotency-associated transcription factors including *Nanog* in Tfe3-ERT2-expressing cells. Only knockdown of *Esrrb* reduced Tam-dependent resistance to differentiation, by approximately 50% (Figure 6D). Likewise, cotransfection of *Esrrb* siRNAs together with *Flcn* or *Fnip1/2* depletion restored commitment (Figure 6E). Conversely, nuclear Tfe3 failed to inhibit exit from the ES cell state in *Esrrb* knockout cells (Figure S6J). These results suggest that *Esrrb* acts epistatically to *Flcn*-*Fnip1/2*-*Tfe3*. We note, however, that knockdown of *Esrrb* alone destabilized self-renewal (Figures 6D and 6E), consistent with its pivotal role in the pluripotency circuit (Nichols and Smith, 2012).

### Nuclear Tfe3 Maintains an ESC State

**Figure 7:**
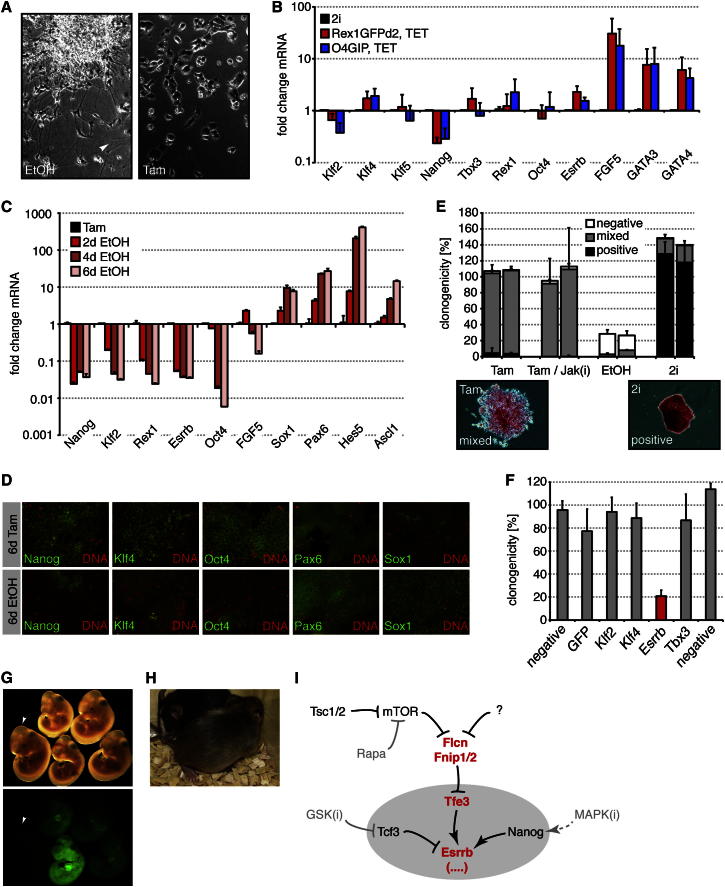
Nuclear Tfe3 Maintains an ESC State (A) Tfe3-ERT2-expressing cells differentiated into neurons (arrowhead) in the absence (left panel) but retained undifferentiated morphology and could be serially passaged in the presence of 0.1 μM Tam (right panel). (B) mRNA expression in Rex1GFPd2 and O4GIP TET cells relative to ESCs in 2i conditions. Average and SD are of three independent experiments. (C) mRNA expression in Rex1GFPd2 TET cells after Tam withdrawal relative to presence of Tam. Average and SD are of two technical replicates. (D) Immunohistochemistry in Rex1GFPd2 TET cells treated for 6 days with and without 0.1 μM Tam. (E) TET cells were plated at single-cell density in indicated culture conditions. Resulting colonies were stained for AP (lower panel) and quantified. Average numbers relative to Tam and SD are from two independent experiments (left bar: Rex1GFPd2, right bar: O4GIP). (F) Rex1GFPd2 TET cells were transfected with indicated siRNAs and replated after 2 days at clonal density in the presence of Tam. Average clonogenicity relative to negative siRNA and SD are of two independent experiments. (G) Rex1GFPd2 TET cells stably transfected with a GFP-expressing plasmid were microinjected into blastocysts, and resulting embryos analyzed at E11.5. Widespread contribution was detected in 4/7 embryos. For comparison, a non-GFP-expressing embryo (arrowhead) of the same litter is shown. (H) Passage 8 Rex1GFPd2 TET cells were cultured for 2 days in 2i and injected into C57BL/6 blastocysts. Contribution of the TET cell *agouti* gene to coat color is visible against black host fur. (I) Schematic representation of the Flcn-Fnip1/2 pathway and combinatorial inputs into *Esrrb* transcription. (….) denotes additional Tfe3 targets that contribute to self-renewal. See also Figure S7.

**Figure S7:**
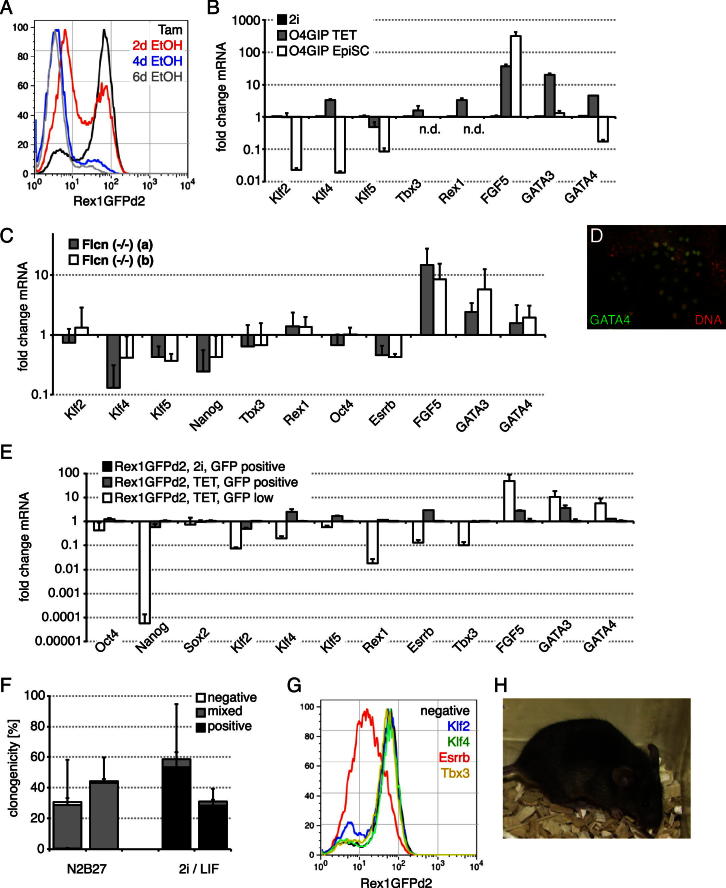
Related to Figure 7 (A) Flow cytometry of Rex1GFPd2 TET cells after Tam withdrawal. (B) Expression levels of indicated mRNAs in O4GIP TET and EpiSCs normalized to 2i. Average and SD are from two independent experiments. (n.d.) indicates not detectable. (C) Expression levels of indicated mRNAs in *Flcn* knockout cells maintained in N2B27 without 2i or LIF normalized to wild-type ESCs in 2i/LIF. Average and SD are of three independent experiments. (D) Immunohistochemistry for GATA4 in Rex1GFPd2 TET cells. (E) Rex1GFPd2 TET cells were sorted for GFP expression and expression of indicated mRNAs determined. Fold changes relative to sorted Rex1GFPd2 ESCs maintained in 2i and SD are from two technical replicates. (F) *Flcn* knockout cells maintained in N2B27 without 2i or LIF were plated at single-cell density in indicated culture conditions. Resulting colonies were stained for AP and quantified. Average relative to number of plated cells and SD are of three independent experiments (left bar: Flcn (−/−) (a), right bar: Flcn (−/−) (b)). (G) Rex1GPPd2 TET cells were transfected with indicated siRNAs and GFP expression monitored by flow cytometry 2 days after transfection. (H) Passage 8 Rex1GFPd2 TET cells were injected into C57BL/6 blastocysts without 2i preculture. Contribution of the TET cell *agouti* gene to coat color is visible against black host fur.

Tfe3-ERT2-expressing ESCs differentiated into neural progenitors and neurons in N2B27 (Figure 7A, left panel). In the presence of Tam, however, many cells resisted differentiation. These cultures acquired stable morphology within 2–4 passages (Figure 7A, right panel) and could be propagated in N2B27 containing Tam for at least 25 passages. Tfe3-ERT2-Tam (TET) cells derived from Rex1GFPd2 and O4GIP ESCs maintained GFP expression in approximately 60%–90% and 90% of cells in the population, respectively (Figure S7A and data not shown). Transcript profiling showed that, with the exception of *Nanog*, TET cells maintained expression of pluripotency genes at levels similar to those in 2i cells (Figure 7B) and were distinct from EpiSCs (Figure S7B). *Flcn* knockout but not wild-type or heterozygous ESCs could be similarly sustained in N2B27 alone, although expression levels of pluripotency genes appeared lower (Figure S7C). The differentiation markers *FGF5*, *GATA3*, and *GATA4* were upregulated in both cases (Figures 7B, S7C, and S7D). To test whether this is because of ongoing differentiation in the cultures, we sorted GFP-high and -low Rex1GFPd2 TET cells. Indeed, GFP-low cells expressed lower levels of pluripotency and higher levels of differentiation markers compared to GFP-high cells (Figure S7E), indicating that TET cell cultures show heterogeneity, similar to ESCs in serum/LIF (Marks et al., 2012).

Withdrawal of Tam caused downregulation of pluripotency gene expression, whereas the neural progenitor markers *Sox1*, *Pax6*, *Hes5*, and *Ascl1* were induced (Figures 7C and 7D). Both *Flcn* knockout cells and TET cells in the presence of Tam self-renewed at single-cell level, generating AP-positive colonies with a skirt of negative cells (mixed) but very few AP-negative, differentiated colonies (Figures 7E and S7F). Addition of a Janus kinase (JAK) inhibitor did not alter colony composition, indicating that LIF-JAK signaling is not required for TET cell self-renewal.

Knockdown of *Esrrb*, but not *Klf2*, *Klf4*, or *Tbx3*, in Rex1GFPd2 TET cells caused downregulation of GFP expression (Figure S7G), and replating at clonal density showed impaired self-renewal of *Esrrb*-depleted cells (Figure 7F). This is further evidence that *Esrrb* is specifically required for TET cell self-renewal. In this regard, it is also noteworthy that TET cells self-renew without GSK3 inhibition.

Plating TET cells in 2i conditions yielded similar numbers of colonies compared to the presence of Tam, but the majority were undifferentiated and homogeneously positive for AP (Figure 7E). This suggests that the TET cell state is fully responsive to conversion into the naive ground state.

To confirm that TET cells retain embryonic identity and developmental potential in vivo, cells kept in Tam for six passages were stably transfected with a GFP-expression plasmid, sorted for high GFP and after two further passages in Tam injected into mouse blastocysts. Resulting embryos showed widespread contribution of GFP-positive cells at E11.5 (Figure 7G). Furthermore, two independently generated TET cell lines contributed to adult tissues. Extensive coat color chimerism was obtained with or without pretreatment in 2i in 4/5 or 4/9 and 6/7 or 2/7 mice, respectively (Figures 7H and S7H and data not shown).

## Discussion

Exit from the ESC state requires dissolution of the core pluripotency transcription-factor circuit, but how the differentiation machinery breaks down this network is unclear. Recovery of several activators in the GSK3 and MAPK pathways (Figure 1D) indicated that our screen was effective in identifying drivers of commitment. GSK3, via β-catenin and Tcf3, represses the orphan nuclear receptor *Esrrb*, a central member of the pluripotent circuitry (Festuccia et al., 2012; Ivanova et al., 2006; Martello et al., 2012; Percharde et al., 2012). Besides *Tcf3* and the GSK3-scaffold gene *APC*, we recovered *CtBP2*, a Tcf3 interactor that may act as a transcriptional corepressor (Brannon et al., 1999; Tarleton and Lemischka, 2010). Autocrine FGF4 signaling drives ESC differentiation, and we recovered two kinases, *KRas* and *Raf1*, in this pathway as well as the MAPK-scaffold protein *Mapksp1/Mp1* (Westerman et al., 2011). Mp1 is required for MAPK signaling at late endosomes (Teis et al., 2002), and we also identified *Hgs/Hrs*, an ESCRT-0 complex member important for FGF receptor endocytosis and signaling (Chanut-Delalande et al., 2010).

We found that efficient exit from naive pluripotency requires components not previously implicated in ESC biology, namely *Flcn*-*Fnip1/2* and the mTOR regulators *Tsc1* and *Tsc2*, that converge on relocalization of the bHLH transcription factor Tfe3. Conversely, enforced nuclear Tfe3 prevents commitment and maintains an ESC state. Interestingly, *Tsc1/2* and *Flcn* are tumor suppressors, whereas *Tfe3* is a protooncogene. *Tfe3*-gene fusions can be causal for renal cell carcinomas, which also occur in tuberosclerosis due to mutations in *Tsc1* or *Tsc2* and in Bird-Hogg-Dubé (BHD) syndrome due to *Flcn* mutations (Armah and Parwani, 2010; Linehan et al., 2010). There are differences in the histopathological classifications of these tumors, but there may be a common underlying pathway. Consistent with this, nuclear Tfe3 has been described in BHD kidney cancers (Hong et al., 2010).

### The Flcn Pathway

The primary sequence of Flcn does not feature recognizable domains. A Flcn protein complex purified from HEK293 cells contained Fnip1 and AMP kinase (AMPK) (Baba et al., 2006). Fnip2 has been identified based on sequence homology and shown to bind to Flcn, Fnip1, and AMPK (Hasumi et al., 2008; Takagi et al., 2008). Although we only identified Fnip1 in Flcn IPs, simultaneous knockdown of *Fnip1* and *Fnip2* was required for pronounced commitment defects. This may be due to redundancy or a Flcn-independent function of Fnip1/2, but the similarity in phenotypes between *Flcn*- and *Fnip1/2*- depleted ESCs is consistent with them acting in the same pathway. Whereas absence of *Flcn* impedes embryogenesis (Hasumi et al., 2009), *Fnip1*-deficient mice are viable (Baba et al., 2012; Park et al., 2012), suggesting redundancy with *Fnip2*. However, *Fnip1* and conditional *Flcn* mutants show similar defects in B cell development (Baba et al., 2012), indicating that requirement for *Fnip2* is facultative and cell type dependent.

Depletion of *Tsc1* or *Tsc2,* similar to *Flcn*-*Fnip1/2*, induced nuclear accumulation of Tfe3 and impaired exit from pluripotency. Epistasis experiments with rapamycin and Tfe3 are consistent with mTOR acting upstream of and/or in parallel with Flcn-Fnip1/2 to inhibit Tfe3-dependent ESC commitment. Phosphorylation of Flcn downstream of mTOR has been described (Baba et al., 2006; Piao et al., 2009), suggesting a linear relationship between Tsc1/2, mTOR, and Flcn-Fnip1/2 (Figure 7I). However, the identity of the kinase and how phosphorylation affects Flcn remains to be determined. In vivo, *Tsc1* and *Tsc2* knockout mice show defects after gastrulation at around E10.0 (Kobayashi et al., 1999, 2001), whereas *Flcn* mutants are affected by E6.0 (Hasumi et al., 2009). *Tsc* mutant embryos feature phenotypic variability and developmental delay, though, which may suggest earlier defects that are compensated for by regulative mechanisms.

We postulate that *Tsc1/2* and *Flcn*-*Fnip1/2* phenotypes in ESCs are mediated by nuclear translocation and activation of Tfe3 (Figure 7I). Depletion of Tfe3 accelerated ESC commitment, indicating that Tfe3 resists breakdown of the pluripotency network. However, *Tfe3* mutant mice are viable and fertile (Steingrimsson et al., 2002). MiTF, Tfe3, and Tfeb can interact with each other (Hemesath et al., 1994), and functional redundancy has been reported (Huan et al., 2006; Steingrimsson et al., 2002). In fact, although *Tfeb* was not essential for *Tsc2* or *Flcn*-*Fnip1/2* phenotypes (Figures S5A and 6E), its overexpression impaired differentiation (data not shown). Thus, redundancy could explain why *Tfe3* mutants have no overt developmental phenotype. It should also be noted that several factors important for in vitro ESC self-renewal are dispensable in the embryo (Nichols and Smith, 2012).

### Regulation of Pluripotency

However, early lethality of *Flcn* mutant embryos is consistent with a role of the Flcn-Fnip1/2-Tfe3 module in progression of pluripotency in vivo. We observed a change in subcellular localization of Tfe3, suggesting that Flcn-Fnip1/2 is inactive in the early ICM and then becomes active in epiblast but not hypoblast cells. *Flcn*, *Fnip1, Tsc1*, and *Tsc2* mRNA levels persisted during ESC differentiation (Figure 3E) and the transition from ICM to epiblast cell identities (Tang et al., 2010). Furthermore, Flcn-Fnip1 complex stoichiometry was maintained during exit from the ESC state (Figure 3D), suggesting posttranscriptional regulation.

Nuclear exclusion of Tfe3 in postimplantation epiblast cells appears to coincide with epithelialization prior to gastrulation and loss of ESC derivation potential (Nichols and Smith, 2011). Tfe3 similarly translocates into the cytoplasm at the onset of ESC differentiation and is cytoplasmic in EpiSCs. These observations suggest that the Flcn-Fnip1/2-Tfe3 module acts as a cell-fate switch that steers naive early epiblast and ESCs into a primed postimplantation state in vitro and in vivo.

Flcn-Fnip1/2-Tfe3 activity requires an ESC-specific context, as nuclear translocation of Tfe3 or knockdown of *Tsc2* and *Flcn*-*Fnip1/2* in EpiSCs did not induce naive pluripotency. Similarly, ectopic activation of Tfe3 efficiently impeded ESC differentiation in the first 24 hr after inhibitor withdrawal, but less so afterward (Figure 5E).

The ability of nuclear Tfe3 to maintain an ESC state in the complete absence of pro-self-renewal or antidifferentiation signals is unprecedented and implies potent input to the transcriptional circuitry sustaining self-renewal (Nichols and Smith, 2012). Functional analysis indicates that transcriptional upregulation of *Esrrb* is a relevant output of nuclear Tfe3. It is likely that there are additional Tfe3 targets because TET cells self-renew without requirement for either MAPK or GSK3 inhibition, whereas forced expression of Esrrb confers independence only from the latter (Martello et al., 2012). Nonetheless, it is striking that transcription of *Esrrb* is regulated by Flcn-Fnip1/2-Tfe3, GSK3/β-catenin/Tcf3 (Martello et al., 2012), and Nanog (Festuccia et al., 2012) (Figure 7I). We surmise that repression by Tcf3 combined with withdrawal of Tfe3 and downregulation of Nanog act to ensure prompt and efficient silencing of this pivotal pluripotency factor at the onset of differentiation. It remains to be tested whether distinct regulatory inputs act in parallel or in sequence, but it is striking that absence of *Tcf3* does not infringe development prior to gastrulation (Merrill et al., 2004), suggesting compensatory dependence of *Esrrb* on Nanog or Tfe3 in vivo.

In summary, this study has identified a module that was previously undescribed in ESCs and mediates a switch from naive to primed pluripotent states. Our findings suggest that nuclear exclusion of Tfe3 acts as a rheostat for controlled disequilibration of the regulatory network that establishes ground-state pluripotency in the embryo and sustains ESCs in vitro.

## Experimental Procedures

Full details are provided in the Extended Experimental Procedures.

### Mice and Embryos

Chimeras were generated by microinjection into C57BL/6 blastocysts. Animal experiments were approved by the University of Cambridge Ethical Review Committee and authorized by the UK Home Office.

### Cell Culture

ESCs were maintained in serum-free N2B27 medium containing 2i and, where indicated, LIF (Wray et al., 2011; Ying et al., 2008). EpiSCs were cultured as described (Guo et al., 2009).

### Commitment Assay

ESCs were plated on gelatin-coated plates at 1.5 × 10^4^ cells/cm^2^. The next day, cells were washed with PBS (PAA) and medium changed to N2B27 without 2i or LIF. Commitment of O4GIP ESCs was determined by restoring 2i plus 1 μg/ml puromycin (Sigma). Uncommitted ESCs were quantified after 2–3 days by adding Alamar Blue (Invitrogen), diluted 1:10 in 2i medium, and read out on a SpectraMax M2 (Molecular Devices). Commitment of other genotypes was quantified by replating on laminin-coated plates in ESC medium. For Rex1GFPd2 cells, 10 μg/ml blasticidin (PAA) was added to select for Rex1 expression.

### siRNA Transfection

Cells were reverse transfected with 16.7 nM siRNA in 2i or FGF2/Activin A containing N2B27 medium with transfection mixes prepared with RNAiMax or Lipofectamine 2000 (Invitrogen) in OptiMEM (Invitrogen). All siRNAs with the exception of Tcf7l1/Tcf3 (Dharmacon, L-048614-01-0005) and Tbx3 (Ambion, 223884 and 223885) were purchased from QIAGEN (FlexiTube GeneSolution siRNA pools; AllStars Negative Control siRNA, 1027281; GFP-22 siRNA, 1022064). Knockdown of *Tcf3* is included as a reference.

Extended Experimental Procedures **Cell Culture**

ESCs were cultured on plastic coated with gelatine or laminin (Sigma). Medium was N2B27 (NDiff N2B27 base medium, Stem Cell Sciences Ltd.) supplemented with small-molecule inhibitors PD03 (1 μM, PD0325901), CHIR (3 μM, CHIR99021). Where indicated, 10 ng/ml LIF (prepared in-house), 4-hydroxytamoxifen (0.1 μM, Sigma), rapamycin (20 nM, Calbiochem), and JAK inhibitor I (10 μM, Calbiochem) were added. *Esrrb* knockout and overexpressing ESCs have been described (Martello et al., 2012). *Flcn* mutant ESCs were derived from intercrossed *Flcn* flox/+ mice, genotyped as described (Hasumi et al., 2009) and CreERT2-expressing clones of one wild-type, one heterozygous, and two homozygous cell lines (denoted (a) and (b)) established in N2B27 supplemented with 2i and LIF. For alkaline phosphatase assays (Sigma), cells were grown on laminin-coated plates, fixed, and stained according to the manufacturer’s instruction. O4GIP-7 (Guo et al., 2009), OEC-2, and EpiSCs expressing the GY118F chimeric LIF receptor (Yang et al., 2010) were cultured on Fibronectin (Millipore)-coated plates with N2B27 supplemented with 12 ng/ml FGF2 and 20 ng/ml Activin A (prepared in-house).

**EpiSC Reprogramming**

EpiSCs were plated at 1.5 × 10^4^ cells/cm^2^. The next day, medium was changed to 2i and, if indicated, supplemented with 30 ng/ml GCSF (Peprotech). After 4 days, medium was changed to 2i, and 2 days later, 1 μg/ml puromycin was added. Reprogramming was quantified by cell survival using Alamar Blue or counting alkaline phosphatase-positive colonies.

**siRNA Screen**

Transfection mixes containing 0.25 μl RNAiMax in 50 μl OptiMEM in gelatin-coated 96-well plates were mixed with 5 μl of 0.5 μM siRNA pools using a pipetting robot (NanoScreen NSX-1536). One hundred microliters of a 5 × 10^4^/ml O4GIP ESC solution in 1.5× concentrated 2i in N2B27 was dispensed in each well using a semi-automated cell dispenser (Genetix Cell Dispense). The next day, cells were washed once with PBS and differentiation induced by changing medium to N2B27. After 72 hr, medium was changed to 2i containing 1 μg/ml puromycin and, 48 hr later, 2i medium containing puromycin and 1/10 vol Alamar Blue (Invitrogen). Cell survival was quantified on a BioTek Flx800 microplate reader. Each 96-well plate contained 11 wells transfected without siRNA that were used for normalization within each plate. We used the mouse druggable genome release 1 and a customized transcription factor siRNA library (QIAGEN) designed against 8,296 and 1,640 genes, respectively. The screen was performed in experimental duplicate and Z scores determined for each run (R^2^ = 0.483). Hits with Z > 3 and Z > 2.5 in the two trials, equaling to less than a 1% probability being false positive, were selected for further analysis. Genes eliminated manually were related to mitochondrial metabolism (Hccs, Mrps12, Cox6c, Uqcrc1, Cox4l1, Ndufv1, Uqcrc2) possibly involved in puromycin-dependent cell death. For validation, candidate siRNAs were reordered (QIAGEN) and retested as siRNA pools and individually.

**Gene-Expression Analysis**

Total RNA was isolated using QIAshredder and RNeasy Kit (QIAGEN), and cDNA synthesized using SuperScriptIII (Invitrogen) and oligo-dT primers. For real-time PCR, we used TaqMan Fast Universal Master Mix and TaqMan probes (Applied Biosystems) or the Universal Probe Library (UPL, Roche) system. Primer sequences and UPL probe numbers are detailed in Table S2. An endogenous control (GAPDH, Applied Biosystems) was used to normalize expression.

**Flow Cytometry**

Live ESCs were resuspended in the presence of 0.05 nM ToPro-3 (Invitrogen) to detect dead cells. Flow cytometry analyses were performed using a CyAn ADP flow cytometer (Dako) and evaluated using FlowJo software. Cell sorting was performed on a MoFlo (Dako).

**ChIP-Seq**

ESCs were fixed for 10 min in 1.1% formaldehyde, neutralized with glycine, collected in cold PBS, and incubated for 10 min on ice in swelling buffer (50 mM HEPES, pH 7.5, 140 mM NaCl, 1 mM EDTA, 10% glycerol, 0.5% NP-40, and 0.25% Tx-100). Nuclei were pelleted, washed, and resuspended in shearing buffer (50 mM Tris, pH 8.0, 10 mM EDTA, and 1% SDS). Lysates were sonicated using a Bioruptor (Diagenode). Lysates were diluted 1:10 in ChIP dilution buffer (50 mM Tris, pH 8.0, 167 mM NaCl, 1.1% Tx-100, and 0.11% Na-Deoxycholate), precleared for 2 hr over ProteinG sepharose beads (Amersham) and incubated overnight with 2 μg Tfe3 (Santa Cruz, sc-5958) or isotype IgG antibodies (Santa Cruz, sc-2025). Lysates were then incubated for 1 hr with blocked ProteinG sepharose beads, washed six times, and eluted twice with elution buffer (1% SDS and 0.1M NaHCO_3_) for 15 min shaking at room temperature. Samples were incubated overnight at 65°C to reverse cross-linking and purified using QIAquick PCR Cleanup kit (QIAGEN). ChiP-Seq libraries were generated using NEBNext ChIP-Seq Library Prep Master Mix Set for Illumina (NEB) using 10 amplification cycles. Thirty-six nucleotide reads were aligned with Bowtie (http://bowtie-bio.sourceforge.net) to the mouse reference genome (UCSC *mm9/NCBI* build 37) excluding mismatches and nonunique mappings. Potential PCR duplicates were removed. Sets of uniquely aligned reads were randomly sampled to equal size, including IgG and *Tfe3* shRNA knockdown controls. Each sample contained 14,524,608 uniquely aligned reads. Peaks were called over IgG and *Tfe3* knockdown controls using MACS (http://liulab.dfci.harvard.edu/MACS), and peaks with at least 100-fold enrichment over IgGs were selected for further analysis. The average peak width of Tfe3 bound regions was 592.2 and 677.6 nucleotides in control and *Flcn* shRNA cells, respectively. ChIP-Seq data sets for Oct4, Nanog, Sox2, Tcf3 (GSE11724) (Marson et al., 2008), and n-Myc (GSE11431) (Chen et al., 2008) were downloaded from GEO. Reads were aligned with Bowtie and peaks called with MACS (FDR < 0.01). Genomic regions ± 25 kb of any gene were split into 1000 bp bins and transcription-factor-binding sites per bin were represented as a binary matrix (Martello et al., 2012). The association of a transcription factor with a gene was further evaluated by the distance-based function sum(exp(-d(i)/5kB)), where the sum was performed over all transcription factor peaks i with a distance d(i) to TSS. The matrices of transcription factors and 1000 bp binding regions/genes were then hierarchically clustered with Pearsson correlation as a distance measure using custom R scripts. Clustering of binding sites was as described (Martello et al., 2012). For motif search, the top 2,000 peak regions were masked for repeats, and regions ± 250 nt around peak summits were scanned for DNA sequence motifs with HOMER (http://biowhat.ucsd.edu/homer). Differences in counts per peak region were evaluated using edgeR (Bioconductor). Microarray data comparing embryonic stem cells and embryoid bodies at 11 time points obtained from GEO (GSE2972) (Hailesellasse Sene et al., 2007) was processed and normalized by RMA using limma (Bioconductor).

**Immunostaining**

Cell and embryo stainings were performed as described (Martello et al., 2012; Nichols et al., 2009). Images were processed using Adobe Photoshop. For automated image quantification, images were acquired using identical parameters on a Leica SP5 confocal microscope and analysis performed using CellProfiler (Broad Institute): DAPI-labeled nuclei and cells were identified, and average fluorescence intensity of nuclei and cytoplasm (cell-nuclei) was determined. Primary antibodies were GATA4 (Santa Cruz, sc-1237, 1:500), Klf4 (R&D, AF3158, 1:500), Nanog (eBiosciences, 14-5761-80, 1:500), Oct4 (Santa Cruz, sc-5279, 1:100), Pax6 (DHSB, 1:50), Sox1 (Cell Signaling, 4194, 1:400), and Tfe3 (Sigma, HPA023881, 1:300).

**Protein Methods**

For immunoprecipitation, ESCs were washed with PBS, incubated on ice in lysis buffer (50 mM Tris, pH 7.4, 150 mM NaCl, 0.5% NP40, 10% glycerol, 1 mM EDTA, 5 mM MgCl_2_, 1 mM DTT) supplemented with Complete Mini protease and PhosSTOP phosphatase inhibitors (Roche), and lysed using a 0.8 mm needle. Lysates were cleared by centrifugation and incubated with FLAG antibodies (Sigma, F1804) and ProteinG sepharose beads for 1 hr. Beads were washed six times with lysis buffer. For mass spectrometry, FLAG immunoprecipitates were eluted twice with 0.4 mg/ml FLAG peptide (Sigma, F4799) in lysis buffer. Elutions were separated by SDS-PAGE, visualized by silver staining, gel tracks sliced into equal segments, in-gel-digested with trypsin, and peptides were analyzed by LC-MS/MS using an LTQ Orbitrap Velos MS (ThermoFisher). Proteins were identified using Mascot (Matrix Science). In two independent experiments, only Flcn (50/42 peptides, Mascot-Score: 994/1561) and Fnip1 (12/4 peptides, Mascot-Score: 598/64) were specifically identified in 3×FLAG Flcn but not control purifications. Nucleocytoplasmic fractionations were performed using a NE-PER Nuclear and Cytoplasmic Extraction Kit (Thermo Scientific) following the manufacturer’s instructions. Cell lysates for western blotting were generated by resuspending cells in RIPA buffer (50 mM Tris, pH 7.4, 150 mM NaCl, 1 mM EDTA, 1% Tx-100, 0.1% SDS) and cleared by centrifugation. Primary antibodies were Flcn (1:100) (Baba et al., 2006), Fnip1 (Novus Biologicals, NBP1-00572, 1:100), GAPDH (Sigma, G8795, 1:1000), Oct4 (Santa Cruz, sc-5279, 1:500), Tfe3 (Sigma, HPA023881, 1:1000), pS6K1 (S371) (Cell Signaling, 9208, 1:100), S6 (Cell Signaling, 2217, 1:100), pS6 (S235/236) (Cell Signaling, 4858, 1:100), and p4EBP1 (T37/46) (Cell Signaling, 2855, 1:100).

**Molecular Biology**

Stable shRNA knockdown clones were generated using OpenBiosystems plasmids (*Flcn*: TRCN0000077487, *Tfe3*: TRCN0000084669). Control cells were generated using empty pLKO.1 vector. Coding sequences were cloned from ESC cDNA using primers containing Gateway (Invitrogen) sites and recombined into pDONR221. To generate shRNA-resistant *Flcn* rescue constructs, silent point mutations were introduced by PCR. For the ERT2-fusion protein, pDONR221-Tfe3 was linearized by PCR and recombined with ERT2 using In-Fusion (Clontech). pDONR221 constructs were recombined into a pPyCAG-IRES-Puromycin-resistance destination vector (for Tfe3-ERT2 expression in *Esrrb* knockout cells), a pPyCAG-IRES-Blasticidin-resistance destination vector (for Tfe3-ERT2 expression in EpiSCs) or PiggyBac destination vectors containing a pgk-hph selection cassette and a CAG-promoter to drive transgene expression with and without an N-terminal 3×FLAG epitope. To generate stable cell lines, PiggyBac constructs were cotransfected with PiggyBac transposase. For control cells, empty vectors without Gateway recombination cassettes were used. For stable GFP expression, TET cells were transfected with pPyCAG-EGFP-IRES-puromycin-acetyltransferase.

## Accession Numbers

ChIP-Seq data sets have been deposited in the GEO database with the ID number GSE39815.

## Supplemental Information

Table S1. List of Tfe3-Bound Regions and Overlap with Nanog, Oct4, Sox2, Tcf3, and n-Myc, Related to Figure 6

Table S2. UPL Primer Pairs Used for qPCR, Related to Experimental Procedures

Document S1. Article plus Supplemental Information
